# Chapin A. Harris, M. D., D. D. S.

**Published:** 1893-04

**Authors:** Samuel J. Cockerille

**Affiliations:** Washington, D. C.


AMERICAN JOURNAL

—OF—

DENTAL SCIENCE.

VOL. XXVI. BALTIMORE, APRIL, 1893. No. 12.

ARTICLE I.

### CHAPIN A. HARRIS, M.D., D.D.S.

BY SAMUEL J. COCKERILLE, D.D.S., WASHINGTON, D. C.

Read before the Virginia State Dental Association, 1892.

With the profession of dentistry there has been no recognition—no proper recognition—of the great and invaluable services of the grandest name ever connected with dentistry. We have waited, hoping it would not fall to the lot of so humble a personage as myself to call attention to this fact. Mr. President, with your consent and that of your Society, we would like to offer a few words to show why this should be done.

The organs with which we have to deal give more pain, cause more suffering, and undermine more constitutions than all other causes put together; we do not except that arch-enemy of the human family—cold. It is our province to prevent this, and keep the economy in its normal condition. Our profession enables us to do this more successfully than any other. A profession is great, grand, noble, and glorious, only in proportion as she can confer benefits upon the human family—a profession is of value *only* in proportion to the amount of good she can accomplish. The study of dentistry as a science does more to quicken the mind and elevate the soul than any other study. Dentistry is the greatest, grandest, noblest, and most glorious profession known to man. To whom are we most indebted for our profession? Dentistry was a dismembered and headless body cumbering the earth.

God made the opportunity, the genius of Chapin A. Harris conceived the idea of a Dental College; and with the aid of other noble spirits, the members were united into a body and a head erected on the body—the Baltimore College of Dental Surgery.

They breathed into it the breath of life and gave to us a profession. When it was our privilege and good fortune to be a student in those halls, the Faculty equaled that of any other institution of learning in this country. We had Cone, Bond, Handy, Pigott, Austen, and the father of our profession. Let no dentist forget that in the dark days of dentistry Chapin A. Harris was the lamp that let light into that darkness.

Books are the real sources of all recorded learning. They chronicle the discoveries in science and perpetuate truth. When the truths they contain were expounded and the discoveries they commemorate were illustrated by these wise and gifted men, we truly felt that no more royal road to learning could be devised, and the young man who sighed for other channels of instruction might well despair of attaining excellence.

The time arrived when dentistry demanded an honorable place among her sister sciences, and the Baltimore Dental College sprang into existence as the natural fruit of Harris' earnest and protracted labors. Previous to this, the study to obtain knowledge of dentistry was mere groping in the dark. When all others practicing dentistry had secrets, his well of knowledge was free to all. To all who were athirst he said, come. Whosoever will let him take of my knowledge freely. He was the first to teach that the attainment of excellence in all things connected with dentistry is not only a high moral and personal duty but is likewise an obligation imposed by the community whose bounty is enjoyed. That the obligations we owe to dentistry are the highest considerations which can influence our actions with reference to our duties on earth; and therefore we should engender and diligently cultivate a sentiment of professional pride. These sentiments are worthy of all commendation, and fit companions in the dentist's bosom for these holy influences which awaken there reverence for and devotion to his God.

Professional pride—Harris' bosom, mind, and soul overflowed with it—is an active desire to see our immediate profession respected, prosperous and happy. It has its origin in that tender feeling, which is the strongest instinct of our natures, incites us to nobler actions and induces greater sacrifices than any other impulse of man's bosom. This feeling is the same as that love and affection for parents, sisters, brothers, friends, that is usually found around the family hearth and which when cultivated, clusters eternally around the heart. No profession can flourish where this sentiment is not taught as a principle, cherished as a passion, and made subordinate only to religion, in the ardor with which it glows in the bosom of her sons. It is the efficiency of this sentiment which controls our actions, stimulates high resolves and secures the sacrifice of individual interest to professional good. The strength, beauty and prosperity of our profession, consists in concentrating our actions, thoughts and affections. A generous competition between individuals, in their effort for honorable distinction, is the greatest incentive to success; so does the rivalry among the members, in their contest for pre-eminence in ameliorating the condition of their patients, ensure to each greater progress in the march of improvement. It is the aggregate of character and prosperity thus attained by the several members which imparts to the profession the glory and grandeur of its character. If a noble spirit of emulation existed and operated in our peofession, to animate and direct us on, what limit could human prophecy affix to our professional advancement? Would to God that our beloved profession thrilled from center to circumference with the inspiration of this spirit. Would that we could this day kindle in every dentist's bosom the regenerating spark; for we are here to speak for Harris and dentistry; and if we speak plainly and boldly, it is because all that we are, all that we have, and all that the future has in store for us are hers, and from our perfect and full consciousness of devotion to her institutions and her interests. We impress it as a duty of paramount importance to cultivate the sentiment of professional pride.

This sentiment should be cherished with ardent love and it will imbue you with unfaltering devotion to our profession, her institutions and to one another. Such devotion, and such devotion only, will lead you in the right —to duty. Remember, the time will soon come when the youngest of you will have to say, our

> Way of life is fallen into the sere,
> The yellow leaf; and the impulsive energies, which alone induce you to strive for the good and honor of the profession, and preserve the name of Chapin A. Harris from oblivion, will be nipped by the frosts of age before your affections and sympathies can attain the haven of professional pride, and onward and upward until full justicc is done the father of our glorious profession. Honor his memory; keep it green in your hearts and hand it down to your children and your children's children to the remotest generation, making the profession a blessing to them. We expected the great State of New York to step forward and do justice to her noblest son; she failing, we turned with anxious eye to his adopted State, the State that gave to the world its first Dental College, but we have waited in vain. She seems to have forgotten the great work of Harris in helping to make Baltimore, her great city, the center of medical education.

The profession turns her eye to Virginia to see the first move towards doing his memory justice. After the discovery of America, the best people landed on this continent settled in Virginia, and she has always led in all that is good. Her great son, Patrick Henry, was the first to conceive the idea of the great rebellion. She gave to the great rebellion its leader—the arch-rebel of the world—the immortal Washington. In all branches of learning, art and science, her sons have been the greatest. Time will never efface the fact that Stonewall Jackson, the greatest soldier and purest Christian, and most perfect man of history, and the noble old Roman of the nineteenth century, Robert E. Lee, were Virginians. Let Virginians always remember that the man who did most for the good and benefit of the human family and nothing to its injury, the gifted and world-renowned Maury, was a Virginian.

While claiming Harris as the morning star of dentistry, we do not forget the noble Virginians who labored successfully for dentistry, and to-day we point you to numbers of your society and State, especially the Nestor of Dentistry—the foremost man in Dentistry—your Thackston, as examples of virtue showing what you may become. In Thackston's wisdom we all delight. If those who do not know him would come to Virginia and question him they would return to their friends and say, behold, the one-half of the greatness of his wisdom was not told them; for Thackston's wisdom exceeds the fame they had heard.

After the most terrible war which was waged against the Southern States by the Northern and Western States, the Federal Government recruiting her armies from all quarters of the globe, endurance, courage and generalship succumb to numbers. Virginia bore the brunt of battle, but from the ruin her queenly form rose, and is now the admiration of us all. It must be plain to all why we turn to Virginia asking that justice be done to the memory of our greatest dentist. All hearts by the impulse of an instinct, which is a surer guide than reason itself, turn to Virginia.

Considered simply as a dentist, Harris was a genius. He devoted his life to the earnest study of medical and scientific literature. It would be difficult to overestimate the value of the services which he rendered to the cause of higher education and instruction. Every lover of dentistry owes a distinct debt of gratitude to the man. Happy were those students who sat under his teachings and heard his wisdom; as that which we are we shall teach, not voluntarily, but involuntarily. The more thoroughly disciplined and fit a man may be for any really great work, the more conscious will he be of his own unfitness for it, the more distrustful of himself, the more anxious not to thrust himself forward. How different the zeal of the half-instructed.

His rule of conduct was to keep his mind unbiased by theories and speculations; never to have any wish that an investigation should result in favor of this view in preference to that; and never to attempt by premature speculation to anticipate the results of investigations, but always to trust to the investigations themselves. Satan, clothed in the light of an angel, has blinded the judgment of the profession; but Harris would never have been led astray. Dr Edward Maynard, and a few others; be it said to their credit, were never deceived and led into error by his glittering show. The touch-stone of truth should have an abiding home in the conscience of each and every dentist and be applied to all operations; and remember, that duty is the sublimest word in the English language. If meat maketh my brother to stumble, I will eat no flesh forevermore, that I make not my brother to stumble.

Harris understood that students are very apt to make the conduct and attainments of their teachers a standard for their own standing. Without a watchful eye over our own hearts and heads, we are lead to regard unduly the opinions not of teachers only, but of the world; and this may prompt us to do what an untrammeled conscience would condemn. Therefore, we should abandon all vicious connections. We should not expect to keep entirely free from charlatans and the unprincipled. Circumstances over which we have not control may bring us in contact with persons whose habits and manners we cannot control. Treat them with all the kindness and politeness due from man to man, but do not make them your favorites and bosom friends. We must be determined not to allow such persons to influence our actions and judgment. Is the poor creature worthy to be called man who says he would willing, ay, gladly reform his ways, but that his associates will not permit him? We say to such, be a man; cast off such companions, and follow the dictates of your judgment. If any one allows his principles to be blasted and his conduct controlled and regulated by another to his own detriment, is he not a fool? and does he not forfeit our respect? Alas! how many have ruined their professional hopes by following the wishes and advice of friends whose character they knew would prove hurtful, as they were surely deficient. We know that a man in the wrong feels at times that he would gladly move himself from evil influences, but he does not attempt it. Are we afraid of the ridicule and scoffs of such friends, or is their company so sweet we cannot live without it? How deplorable the condition of him whose enjoyments depend so much upon another, that he cannot part with him at the call of professional pride and duty. Can any right thinking man be ready to barter the good of his profession for the company of him who is a detriment to that profession? Is there no satisfaction in breaking off from those who furnish us only bad examples, and seeking the society of the wise and the good? The example of the wise and the good purifies and strengthens our judgment. In following the company of those who are striving to keep down dentistry, we prove ourselves to be slaves. We comply with our evil passions and call a license liberty. We are tempted to that which is wrong. We follow our low and corrupt propensities and passions and are led into bondage. We are told they are the silken bands of friendship. They may be silken, but they drag down our profession and lower our professional standing. A creature in this unfortunate situation is truly to be pitied.

Harris was a hard worker, and devoted all the resources of his cultivated mind to further the welfare of his profession. He realized the importance of study, education and drill, in the most important art of operating upon the teeth; that it is the duty of a good man to cultivate truth, purity and industry. With the students he was punctual, truthful, straightforward, industrious and always kind. In his family, he was forbearing and loving to his children; to his wife he was the perfection of purity and devotion.

Harris was undoubtedly the most prominent man ever in dentistry, around whom, as the glorious noonday sun, have revolved for half a century the lesser lights in comparative obscurity. Full well do we know it has been the effort of the less gifted to pull down by envious detraction his towering reputation. Thank God, it was built upon such a solid and perfect foundation that no earthly power can shake it. He was more than a dentist—he was a Christian gentleman. Strive to imitate his genius and emulate his virtues. Let not the names of our great and useful men pass into oblivion.

